# Supplementary material for: Detection of SARS-CoV-2 in Wastewater Associated with Scientific Stations in Antarctica and Possible Risk for Wildlife
Source: Microorganisms. 2024 Apr 6;12(4):743. doi: 10.3390/microorganisms12040743 (PMC11051888; doi:10.3390/microorganisms12040743)
Supplement: Supplementary file 1 [file microorganisms-12-00743-s001.zip › microorganisms-2878792-supplementary.pdf]

Suplemmentary table S1: Primers used in this study

	Sequence	Type
N_1F	GACCCCAAAATCAGCGAAAT	Forward
N_1R	TCTGGTTACTGCCAGTTGAATCTG	Reverse
Probe N_1	FAM-ACCCCGCATTACGTTTGGTGGACC-BH1	
N_2F	TTACAAACATTGGCCGCAAA	Forward
N_2R	GCGCGACATTCCGAAGAA	Reverse
Probe N_2	FAM-ACAATTTGCCCCCAGCGCTTCAG-BHQ1	
RNAsaPF	AGATTTGGACCTGCGAGCG	Forward
RNAsaPR	GAGCGGCTGTCTCCACAAGT	Reverse
Probe RNAsaP	FAM-TTCTGACCTGAAGGCTCTGCGCG-BHQ1	
